# Marine *n–3* Long-Chain Polyunsaturated Fatty Acid Intake in Pregnancy and Risk of Early Life Infections in 3 Nordic Cohorts: A HEDIMED Consortium Study

**DOI:** 10.1016/j.tjnut.2026.101456

**Published:** 2026-02-28

**Authors:** Aino K Rantala, Leena Hakola, Nicklas Brustad, German Tapia, Elin M Hård Af Segerstad, Jussi Lehtonen, Jonathan Thorsen, Mari Åkerlund, Christine L Parr, Maria C Magnus, Nicolai A Lund-Blix, Jakob Stokholm, Mikael Knip, Jorma Toppari, Ketil Størdal, Riitta Veijola, Heikki Hyöty, Suvi M Virtanen, Klaus Bønnelykke, Lars C Stene, the HEDIMED Investigator Group

**Affiliations:** 1 Department of Chronic Diseases, Norwegian Institute of Public Health, Oslo, Norway; 2 Center for Environmental and Respiratory Health Research (CERH), Research Unit of Population Health, University of Oulu, Oulu, Finland; 3 Department of Public Health, Finnish Institute for Health and Welfare, Helsinki, Finland; 4 Faculty of Social Sciences, Unit of Health Sciences, Tampere University, Tampere, Finland; 5 Tampere University Hospital, Research, Development and Innovation Center, Tampere, Finland; 6 Copenhagen Prospective Studies on Asthma in Childhood, Herlev and Gentofte Hospital, Copenhagen, Denmark; 7 Department of Paediatric and Adolescent Medicine, Oslo University Hospital, Oslo, Norway; 8 Celiac Disease and Diabetes Unit, Clinical Sciences Malmoe, Lund University, Malmoe, Sweden; 9 Department of Virology, Faculty of Medicine and Health Technology, Tampere University, Tampere, Finland; 10 Department of Clinical Medicine, University of Copenhagen, Copenhagen, Denmark; 11 Norwegian Scientific Committee for Food and Environment, Oslo, Norway; 12 Centre for Fertility and Health, Norwegian Institute of Public Health, Oslo, Norway; 13 The Norwegian Childhood Diabetes Registry, Division of Childhood and Adolescent Medicine, Oslo University Hospital, Oslo, Norway; 14 Department of Food Science, University of Copenhagen, Frederiksberg C, Denmark; 15 Research Program for Clinical and Molecular Metabolism, Faculty of Medicine, University of Helsinki, Helsinki, Finland; 16 Department of Pediatrics, Tampere University Hospital, Tampere, Finland; 17 Institute of Biomedicine, Research Centre for Integrative Physiology and Pharmacology, and Centre for Population Health Research, and InFLAMES Research Centre, University of Turku, Turku, Finland; 18 Department of Pediatrics, Turku University Hospital, Turku, Finland; 19 Department of Paediatric Research, Institute of Clinical Medicine, Faculty of Medicine, University of Oslo, Oslo, Norway; 20 Department of Pediatrics, PEDEGO Research Unit, Medical Research Center, University of Oulu, Oulu, Finland; 21 Department of Children and Adolescents, Oulu University Hospital, Oulu, Finland; 22 Fimlab Laboratories, Tampere, Finland; 23 Center for Child, Adolescent and Maternal Health Research, Tampere University and Tampere University Hospital, Tampere

**Keywords:** infections, dietary fatty acids, pregnancy, childhood, cohort study, MoBa, DIPP, COPSAC

## Abstract

**Background:**

*n–3* (ω-3) long-chain polyunsaturated fatty acids (*n–3* LCPUFAs) have anti-inflammatory effects that may influence immune-mediated diseases.

**Objectives:**

We investigated whether higher maternal pregnancy intake of *n–3* LCPUFA is associated with a lower incidence of infections in young children.

**Methods:**

We used data from 3 Nordic cohorts: the Norwegian Mother, Father and Child Cohort study (MoBa, *n* = 76,026), the Finnish Diabetes Prediction and Prevention Study (DIPP, *n* = 560), and the Copenhagen Prospective Studies on Asthma in Childhood 2010 cohort (COPSAC**_2010_**, *n* = 680). Childhood infections up to age 36 mo were assessed using questionnaires in MoBa, coxsackievirus B 1-6 (CVB1-6) neutralizing antibodies in DIPP, and pathogenic viral PCR identification from acute respiratory episodes in COPSAC_2010_. Maternal *n–3* LCPUFA intake was assessed through validated food frequency questionnaires in MoBa and DIPP, whereas COPSAC**_2010_** used a randomized trial design where pregnant women received fish oil capsules or a placebo.

**Results:**

Higher *n–3* LCPUFA intake was not significantly associated with lower respiratory tract infection (adjusted incidence rate ratio [aIRR]: 0.99; 95% CI: 0.94–1.03) but was associated with a reduced risk of upper respiratory tract infections (aIRR: 0.99; 95% CI: 0.98–0.99) and gastroenteritis (aIRR: 0.96; 95% CI: 0.95–0.98) per g/d up to age 36 mo in MoBa. The DIPP study found no association between *n–3* LCPUFA intake and having ≥1 CVB infection (adjusted odds ratio: 1.74; 95% CI: 0.64–4.72, per g/d). The COPSAC_2010_ trial found no significant effects of the intervention for pathogen-specific respiratory episodes (IRR: 0.86; 95% CI: 0.69–1.07).

**Conclusions:**

This study does not provide consistent evidence that higher maternal *n–3* LCPUFA pregnancy intake reduces the risk of infections in early childhood.

**Clinical Trial Registry:**

This trial was registered as NCT00798226, https://clinicaltrials.gov/study/NCT00798226.

## Introduction

Marine *n–3* long-chain polyunsaturated fatty acids (*n–3* LCPUFA), particularly eicosapentaenoic acid (EPA) and docosahexaenoic acid (DHA), have multiple biological effects, including anti-inflammatory properties that may affect the risk of immune-mediated chronic diseases [1]. Dietary intake of DHA and EPA can counteract the production of arachidonic acid-derived eicosanoids, including prostaglandins and leukotrienes, that contribute to inflammation in asthma and other chronic conditions where inflammation plays a role [1,2]. EPA and DHA are precursors of proresolving mediators, important in regaining homeostasis after inflammation [2]. *n*-3 LCPUFAs are known to cross the placenta [3], and a pregnancy diet may influence both maternal and child health [4].

Besides chronic diseases, there are few studies of how dietary DHA and EPA may affect the risk of infections, and some randomized trials have suggested that such dietary supplements can improve respiratory health and prevent infections in children [[5], [6], [7]]. Since infections have been linked to the pathogenesis of immune-mediated diseases, it is possible that DHA/EPA intake contributes to the risk of immune-mediated diseases by regulating the effects of infections. In a randomized trial of fish oil supplementation during pregnancy, the risk of wheeze and asthma in the child was reduced, compared to capsules with olive oil [8]. Analysis of secondary endpoints further showed that supplementation with *n−3* LCPUFA was also associated with a reduced risk of lower respiratory tract infections (LRTI) [8], the viral croup [9], and gastroenteritis [10].

These findings call for more studies to clarify whether maternal *n–3* LCPUFAs affect specific types of infections in the offspring. The objective of this study was therefore to investigate whether maternal dietary intake of marine *n–3* LCPUFA during pregnancy may influence offspring risk of early childhood infections. We present data from 2 birth cohort studies and a randomized trial aimed at investigating whether higher maternal dietary intake of *n–3* LCPUFA during pregnancy is associated with the incidence of different types of childhood infections that have been linked to the pathogenesis of asthma and/or type 1 diabetes. In the Norwegian Mother, Father and Child Cohort study (MoBa), we tested whether maternal intake of *n–3* LCPUFAs during pregnancy was associated with a lower frequency of infections in childhood. In the Finnish Type 1 Diabetes Prediction and Prevention (DIPP) study, we tested whether maternal intake of *n–3* LCPUFAs was associated with lower frequency of Coxsackievirus B (CVB) infections in the child. In the Copenhagen Prospective Studies on Asthma in Childhood 2010 (COPSAC**_2010_**) trial, we tested whether the previously described reduced risk of LRTI was specific for any of the common pathogens causing LRTIs during acute respiratory episodes.

## Methods

### Study populations

This study used data from participants in 3 Nordic pregnancy or birth cohorts: the Norwegian MoBa study [11], the Finnish DIPP study [12], and the Danish COPSAC**_2010_** trial [13].

MoBa is a population-based pregnancy cohort study conducted by the Norwegian Institute of Public Health. Pregnant women were recruited across Norway around 18 gestational wks from 1999 to 2008, with a 41% participation rate. The cohort includes over 1,14,000 children and parents in Norway [14]. The current study included MoBa children who were still alive and residing in Norway by 2018, with data from the Norwegian Medical Birth Registry, and completed maternal questionnaires at 22 gestational wks, at the child’s age of 6 mo (*n* = 76,026), 18 mo (*n* = 62,951), and 36 mo (*n* = 47,989) (Supplementary Figure 1). The MoBa study was approved by the Regional Committee for Medical Research Ethics of South/East Norway, and the ongoing data collection in MoBa is approved by the Norwegian Health Registry Act.

DIPP is a population-based birth cohort study of children with human leukocyte antigen-conferred susceptibility to type 1 diabetes in Finland [12]. Altogether 6080 DIPP participants born in Tampere or Oulu during 1997–2004 were enrolled for the study of maternal diet and follow-up of type 1 diabetes-associated islet autoantibodies and clinical type 1 diabetes. The current study includes 560 children from a nested case–control selection within the cohort, with 184 case children who turned positive for multiple islet autoantibodies and 376 matched control children, who had available data on pregnancy [15], and serum neutralizing antibodies to 6 CVB serotypes by the age of 36 mo (Supplementary Table 6) [16]. The nested case–control setting was not utilized in this study. The DIPP study was approved by the Ethics Committees of the Northern Ostrobothnia Hospital District and Pirkanmaa Hospital District.

COPSAC_2010_ includes 738 Danish pregnant women from a population-based cohort who were randomized 1:1 to receive 2.4 g of *n–3* LCPUFA (fish oil), or placebo (olive oil), per day from gestational week 24. Their children were followed with longitudinal clinical, daily symptom diaries, and acute care visits when experiencing any sign of troublesome lung symptoms such as cough, wheeze, or dyspnea severely affecting the child during the first 36 mo of life as previously reported [8]. A total of 695 children were included in the *n-3* LCPUFA trial. The trial was conducted in accordance with the guiding principles of the Declaration of Helsinki and was approved by the local ethics committee (H-B-2008-093) and the Danish Data Protection Agency (2015-41-3696). The trial is registered at clinicaltrials.gov (identifier NCT00798226) on November 26, 2008.

### Childhood infections

#### MoBa

The *a priori* primary outcome in MoBa was the mean number of LRTI episodes (pneumonia, acute bronchitis) in the first 36 mo. We also tested upper respiratory tract infection (URTI) (common cold, influenza, croup, otitis media, throat infection) and gastroenteritis episodes up to 36 mo, as well as these outcomes up to ages 6 and 18 mo, and hospitalization for these conditions (Table 1). The number of infections was assessed by parents’ reports in questionnaires when the child was 6-, 18-, and 36-month-old.

**TABLE 1:** Frequency of parental-reported childhood infections in the Norwegian Mother, Father and Child Cohort study

Infection	0–6 mo, *n* = 76,026		0–18 mo, *n* = 62,951		0–36 mo, *n* = 47,989	
	≥ 1 episode *n* (%)	Number of episodes, mean (SD)	≥ 1 episode *n* (%)	Number of episodes, mean (SD)	≥ 1 episode *n* (%)	Number of episodes, mean (SD)
**LRTI**	3631 (4.8)	0.1 (0.3)	9333 (14.8)	0.2 (0.7)	10,686 (22.3)	0.4 (1.0)
LRTI, medical visits	3461 (4.6)		3990 (6.3)		3946 (8.2)	
**URTI**	57,930 (76.6)	1.5 (1.4)	62,223 (98.9)	6.5 (4.0)	47,925 (99.9)	10.8 (5.5)
Croup	1568 (2.1)	0.02 (0.2)	6718 (10.7)	0.2 (0.7)	8900 (18.6)	0.5 (1.4)
Otitis media	3334 (4.5)	0.05 (0.3)	18,697 (29.8)	0.6 (1.3)	21,896 (45.6)	1.2 (2.0)
Throat infections	2249 (3.1)	0.03 (0.2)	12,099 (19.3)	0.3 (0.8)	15,824 (33.0)	0.6 (1.3)
Common cold	57,325 (76.5)	1.5 (1.3)	61,996 (98.5)	5.5 (3.1)	47,886 (99.8)	8.6 (4.0)
URTI, medical visits	19,604 (25.9)		16,778 (26.7)		15,034 (31.3)	
**Gastroenteritis**	8337 (11.0)	0.1 (0.4)	49,829 (63.3)	1.2 (1.4)	41,277 (86.0)	2.5 (2.1)
GE, medical visits	2404 (3.3)		2746 (4.4)		2801 (5.8)	

GE, gastroenteritis; LRTI, lower respiratory tract infection; n, number; URTI, upper respiratory tract infection.

#### DIPP

Information on CVB infections was based on the appearance of neutralizing CVB antibodies in serum samples serially collected during the follow-up, with a median (IQR) of 6 (4, 6) samples per child between ages 1 and 36 mo, averaging 3- to 6-month intervals. Neutralizing antibodies were assessed using classical plaque reduction assays for 6 CVB serotypes [16]. The timing of infections was set to the time of sample in which CVB antibodies were first detected, indicating seroconversion from antibody negativity to antibody positivity (titer 4 or higher with peak titer ≥16 in ≥1 follow-up samples). If there were maternal viral antibodies in cord blood, the child’s titer had to reach zero, and then 16, before an infection in a child could be defined. The incidence of CVB infections at time windows of 0 to 6 mo, 0 to 18 mo, and 0 to 36 mo was assessed. Missing samples were considered as no infection, which may cause a slight underestimation of infection incidence. Infection incidence among 549 children who had a sample by 36 mo of age was 71%, whereas the prevalence of CVB seropositivity at 36 mo was 82% among 273 children.

Having ≥1 CVB infection by age 36 mo was defined as the primary outcome, but we also investigated risk of each serotype (CVB1-6) separately and the number of different CVBs as the secondary outcomes. The same outcomes were studied separately for the ages 6 mo and 18 mo.

#### COPSAC_2010_

Children who experienced troublesome lung symptoms were invited to the COPSAC clinic during acute care visits, where a nasopharyngeal aspirate sample was collected for viral PCR identification. The pathogens analyzed here were the most common viral pathogens, that is, rhinoviruses, respiratory syncytial viruses (RSV), and enteroviruses, as described previously in our original trial publication [17].

### Maternal n–3 LCPUFA intake and status

#### MoBa

Intake of *n–3* LCPUFAs (EPA + DHA g/d) from food and dietary supplements during pregnancy was retrieved from a validated food frequency questionnaire (FFQ) [[18], [19], [20], [21]]. The validated FFQ was used from 2002 (Supplementary Figure 1). The FFQ considered habitual dietary intake from the beginning of the pregnancy and was collected at ∼22 gestational weeks. The FFQ covers 225 foods and drinks, and dietary nutrient intakes were calculated using FoodCalc and the Norwegian food database (version 2005) using standard portion sizes adjusted to the cohort [19], whereas intake of n–3 LCPUFAs from supplements was estimated using an available contemporary database [22]. Participants with an implausible reported energy intake (<4.5 megajoules or >20 megajoules), or an incomplete FFQ (defined as 4 or more blank pages) were excluded [20]. The study includes a subcohort of 976 mothers with available measurements of fatty acids in the phospholipid fraction of nonfasting EDTA whole blood collected at mid-pregnancy [23], also described in detail in Supplemental Methods.

#### DIPP

The intake of *n–3* LCPUFAs (DHA and EPA g/d) from food and supplements during pregnancy was calculated from a validated semiquantitative FFQ concerning the habitual diet in the eighth month of pregnancy [15]. The FFQ contained questions on the frequency and amount of 181 foods and drinks consumed; the type of fat used in cooking, baking, and salad dressing; the extent of home baking; and the use of dietary supplements. These questions were used to calculate individual nutrient intakes based on the Finnish Food Composition Database (Fineli), and the data were analyzed with the in-house software (Finessi) of the Finnish Institute for Health and Welfare, Finland. FFQs with >10 missing items were excluded. The validity and reproducibility of the FFQ data collection after pregnancy to record diet during 8 mo of pregnancy are generally good [15,24].

#### COPSAC_2010_

The double-blinded randomized clinical trial of 700 mother/child pairs randomized pregnant women to receive 2.4 g daily of *n–3* PUFA as fish oil (55% EPA and 37% DHA) or in similarly appearing capsules containing olive oil in the placebo group. Supplementation continued until 1 wk after birth. Investigators and participants remained blinded until the youngest child reached 36 mo, that is, the pathogens were all analyzed during the double-blinded period. The trial has been described in detail previously [8].

### Statistical analysis

#### MoBa

We assessed the association between total maternal intake of *n–3* LCPUFAs (g/day) during pregnancy as a continuous variable and the incidence of infections by applying negative binomial regression models and estimating the incidence rate ratio (IRR). The number of infections was assessed separately at ages 6, 18, and 36 mo. Associations with doctor’s visit or hospitalization for infections were modeled using binomial regression. Multivariable regression analyses were adjusted for potential confounders as described in the Results section. Covariates included maternal characteristics such as age at delivery, country of birth, parity, educational level, smoking during pregnancy***,*** asthma, total energy intake in pregnancy, vitamin D intake from food and supplements during pregnancy, and prepregnancy BMI (in kg/m^2^), as well as the child characteristics sex, year and month of birth, and county of residence. Information on covariates was retrieved from questionnaires and the Medical Birth Registry of Norway. In sensitivity analyses, we further adjusted for breastfeeding (still at 6 mo), preterm birth, birth weight, and the child’s own dietary supplementation with fish oil at 6 and 18 mo, which are potential mediators of the association between *n–3* LCPUFA intake in pregnancy and risk of infections in childhood.

#### DIPP

Associations between maternal intake of *n–3* LCPUFA were similarly modeled as in MoBa, but with logistic regression, and outcomes were having a CVB infection by the age of 6, 18, or 36 mo. The logit–linearity assumption was met. For the number of different CVB infections, we used ordinal logistic regression. Models were adjusted for islet autoimmunity case–control status, sex, birth year, birth month, birth area (Tampere, Oulu), number of siblings, smoking, and prepregnancy BMI.

#### COPSAC_2010_

The incidence of pathogen-specific infection episodes in the COPSAC_2010_ randomized clinical trial data was analyzed using Quasi-Poisson regression models to account for overdispersion of the count data, that is, the variance is greater than the mean of the count distribution of the data. The data were analyzed in separate models for the 3 pathogen-specific infections (enteroviruses, rhinoviruses, and RSV) until age 36 mo, estimating the IRR. We compared risk of specific virus episodes between the supplementation groups (*n–3* LCPUFA compared with olive oil) and included all children with full follow-up until age 3 y. The analysis was an intention-to-treat and included children with complete outcome data until age 3 y (*n* = 680) and was a post-hoc analysis of the original RCT with minimal (2%) loss to follow-up. There were 350 children who underwent a nasopharyngeal sample, whereas the rest of the children (*n* = 330) were included as healthy children. The baseline characteristics of the pregnant women and their children showed that randomization was not biased; similarly, there were no differences in pregnancy endpoints as reported in detail for the original trial [8].

## Results

### MoBa

Background characteristics of MoBa offspring are shown in Supplementary Table 1. The mean number of parent-reported infections in MoBa children was 0.1 (SD: 0.3) LRTIs, 1.5 (SD: 1.4) URTIs, and 0.1 (SD: 0.4) gastroenteritis episode before age 6 mo, and 0.4 (SD: 1.0) LRTIs, 10.8 (SD: 5.5) URTIs, and 2.5 (2.1) gastroenteritis episodes until 36 mo of age (Table 1). The median daily maternal intake of *n–3* LCPUFA from food and supplements during pregnancy was 0.6 (IQR: 0.3–1.1) g/d (Supplementary Table 1). The median proportion of *n–3* LCPUFA in the phospholipid fraction of whole blood collected at mid-pregnancy in the subcohort of 976 mothers was 5.7% (range, 4.7–6.9).

**FIGURE 1:**
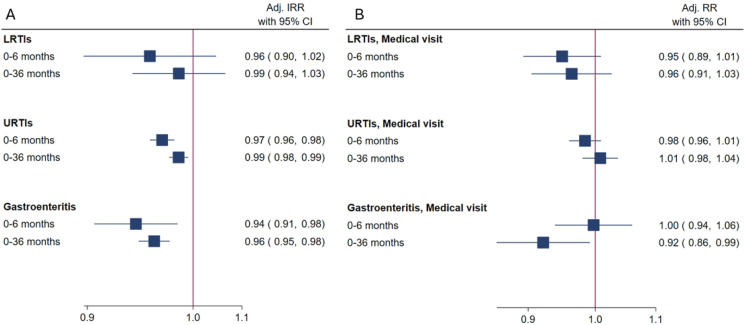
Association of the maternal total intake of *n–3* long-chain polyunsaturated fatty acids (LCPUFA) per g/d in pregnancy with the number of infectious episodes (A) and with doctor visit or hospitalization for infections (B) in the child in The Norwegian Mother, Father and Child Cohort study (MoBa) (*n* = 76,026 at 0–6 mo and *n* = 47,989 at 0–36 mo). Analyses were adjusted for the maternal characteristics, age at delivery, country of birth, parity, educational level, smoking during pregnancy, asthma, total energy intake in pregnancy, vitamin D intake from food and supplement during pregnancy, and prepregnancy BMI, as well as the child characteristic sex, year and month of birth, and county of residence. CI, confidence interval; IRR, incidence rate ratio; LRTI, lower respiratory tract infection; RR, risk ratio; URTI, upper respiratory tract infection.

There was no statistically significant association of daily intake of *n–3* LCPUFA in pregnancy and the risk of LRTIs in the child up to age of 6 (adjusted IRR: 0.96; 95% CI: 0.90–1.02) per g/d and 36 mo (aIRR: 0.99; 95% CI: 0.94–1.03) per g/d (Figure 1A). The *n–3* LCPUFA intake during pregnancy was associated with a lower risk of URTIs in the child up to age 6 (aIRR: 0.97; 95% CI: 0.96–0.98 per g/d) and 36 mo (aIRR: 0.99; 95% CI: 0.98–0.99 per g/d) (Figure 1A). The risk of doctor visits or hospitalization due to LRTIs and URTIs before 6 mo was also lower, but this difference was not statistically significant (aRR: 0.95; 95% CI: 0.89–1.01 and aRR: 0.98; 95% CI: 0.96–1.01, respectively) (Figure 1B) (Supplementary Tables 2 and 3).

We observed an inverse association between the total intake of *n–3* LCPUFA in pregnancy and the number of gastroenteritis episodes in the child up to age 6 (aIRR: 0.94; 95% CI: 0.91–0.98, per g/d) and 36 mo (aIRR: 0.96; 95% CI: 0.95–0.98, per g/d) (Figure 1A). There was also an association between fewer doctor visits or hospitalizations for gastroenteritis and *n–3* LCPUFA intake (Figure 1B). There was no association between *n–3* LCPUFA intake in pregnancy and the risk of croup (Supplementary Tables 3 and 4).

In a sub-analysis, we evaluated the effect of *n–3* LCPUFA separately from food and supplements and found a slight inverse association with LRTIs in relation to *n–3* LCPUFA intake from dietary supplements but not from food (Supplementary Table 2). Further adjustment for the potential mediators, including breastfeeding, preterm birth, birth weight, and the child’s own use of supplements, did not change the associations between maternal *n–3* LCPUFA intake and infection risk (Supplementary Tables 2–4).

In the subcohort of 976 participants, individual variation in the proportion of *n–3* LCPUFA in the phospholipid fraction of whole blood collected at mid-pregnancy was not significantly associated with the number of LRTIs, URTIs, or gastroenteritis in the child before age 36 mo (Supplementary Table 5).

### DIPP

**FIGURE 2:**
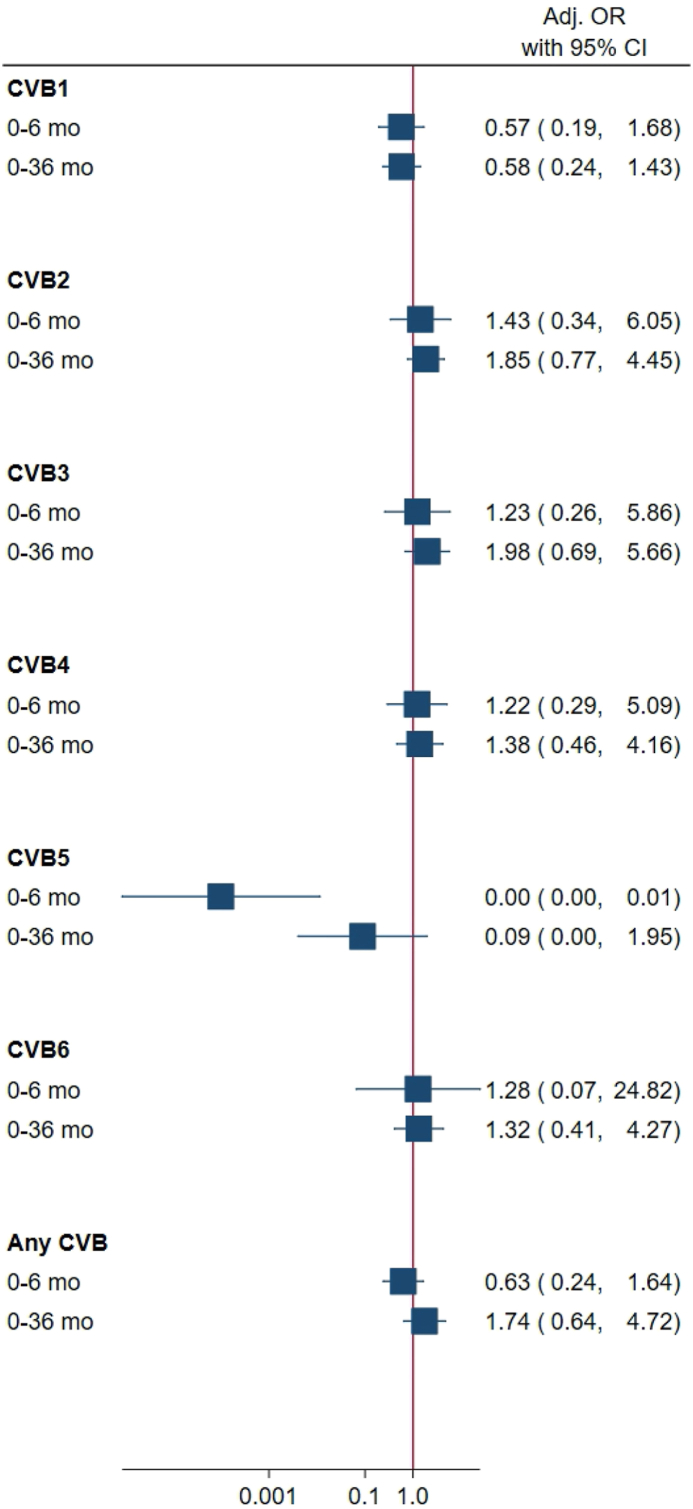
Maternal *n–3* long-chain polyunsaturated fatty acids (LCPUFA) intake during pregnancy and risk of ≥1 coxsackievirus B (CVB) infection in children in the DIPP study. The total *n–3* LCPUFA per g/d in pregnancy was modeled as a continuous exposure in relation to CVB infection in children by the age of 6 and 36 mo. Analyses were adjusted for islet autoimmunity case–control status, sex, birth year, birth month, birth area, number of siblings, smoking, and prepregnancy BMI. Numbers of children varied from 486 to 558 for the different outcomes and age groups. Additional details, including unadjusted associations, are shown in Supplementary Table 7. CI, confidence interval; DIPP, The Finnish Type 1 Diabetes Prediction and Prevention study; OR, odds ratio.

Characteristics of the study population are shown in Supplementary Table 6. The median maternal total daily intake of *n–3* LCPUFA from food and supplements (g/d) during pregnancy was 0.3 (IQR: 0.2–0.4). A total of 12 mothers (2.1%) reported *n–3* LCPUFA intake from dietary supplements. CVB antibodies were common, and 41% of children had ≥ 1 CVB infection by the age of 6 mo, 63% by 18 mo, and 71% by 36 mo (Table 2). The proportion of children having ≥ 2 different CVB infections was 11% by 6 mo, 26% by 18 mo, and 33% by 36 mo of age (Table 2). Overall, the maternal intake of *n–3* LCPUFA in pregnancy was not associated with the risk of any CVB infections in the child by the age of 6, 18, or 36 mo (Figure 2 and Supplementary Table 7). We observed an inverse association between *n–3* LCPUFA intake during pregnancy and CVB5 infection during the first 6 mo of life (Figure 2). When rescaled per 1 mg/day increase in *n–3* LCPUFA intake, the adjusted odds ratio was 0.988 (0.979, 0.998), *P* = 0.018; Supplementary Table 7). The maternal intake of *n–3* LCPUFA in pregnancy was not associated with the number of CVB infections in the child (Supplementary Table 8).

**TABLE 2:** Frequencies and proportions of coxsackievirus B (CVB) infections by type and number of different CVBs by the age of 6, 18, 36 mo in the DIPP study

	0–6 mo			0–18 mo			0–36 mo		
Type of CVB infection									
	No, *n*	Yes, *n*	Yes, %	No, *n*	Yes,*n*	Yes, %	No, *n*	Yes, *n*	Yes, %
CVB1	375	147	28.2	319	237	42.6	301	257	46.1
CVB2	443	46	9.4	369	127	25.6	376	173	31.5
CVB3	485	38	7.3	491	65	11.7	483	75	13.4
CVB4	480	44	8.4	488	68	12.2	480	78	14.0
CVB5	485	11	2.2	509	20	3.8	524	28	5.1
CVB6	478	9	1.8	490	33	6.3	484	65	11.8
Any CVB	287	199	40.9	192	331	63.3	157	392	71.4

Number of different CVB infections									
		*n*	%		*n*	%		*n*	%

0		287	59.1		192	36.7		157	28.6
1		144	29.6		196	37.5		213	38.8
2		33	6.8		89	17.0		116	21.1
3		11	2.3		24	4.6		32	5.8
≥4		11	2.3		22	4.2		31	5.6

CVB, coxsackievirus B; n, number.

### COPSAC_2010_

**FIGURE 3:**
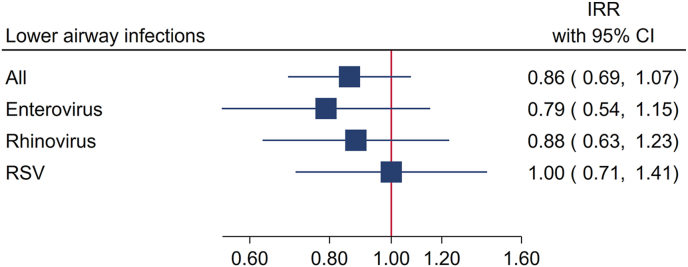
Effect of maternal fish oil supplementation during pregnancy on the frequency of infections in children between age 0 and 36 mo in the Copenhagen Prospective Studies on Asthma in Childhood 2010 (COPSAC**_2010_**) randomized clinical trial. CI, confidence interval; IRR, incidence rate ratio; RSV, respiratory syncytial virus.

Of the 700 mothers, 680 mother–child pairs were included in the study, with acute respiratory virus samples analyzed from the children and mothers included in the randomized clinical trial and with full follow-up during the first 3 y of life. We analyzed effects of prenatal fish oil supplementation (*n* = 340) compared with placebo (*n* = 340) using a Quasi-Poisson regression model and found no significant effects for pathogen-specific respiratory episodes up to 36 mo (enterovirus: IRR: 0.79; 95% CI: 0.54–1.15, rhinovirus: 0.88; 0.63–1.23, and RSV: 1.00; 0.71–1.41) (Figure 3). When analyzing the sum of all 3 respiratory pathogens in a combined variable, there were no differences between the supplementation groups, although the estimate was pointing toward a reduced risk in the intervention group: 0.86 (0.69–1.07).

## Discussion

Findings from the large MoBa study indicate that maternal *n–3* LCPUFAs during pregnancy were associated with a modestly decreased risk of URTIs and gastroenteritis up to 36 mo, but no significant associations were found with LRTIs. In the DIPP study, we found no significant associations of maternal *n–3* LCPUFAs with CVB infections assessed by serology in the offspring. In the COPSAC**_2010_** randomized controlled trial, prenatal fish oil supplementation did not impact the risk of enterovirus-, rhinovirus-, or RSV-positive respiratory episodes.

### Synthesis with previous literature

There is some evidence suggesting prenatal nutrition, particularly maternal intake of *n–3* LCPUFA, may influence childhood infection susceptibility. Experimental studies have shown that *n–3* LCPUFA can inhibit replication of certain viruses, including influenza A virus [25], as demonstrated in mice [26]. In the original COPSAC**_2010_** fish oil trial, supplementation from gestational week 24 until 1 wk postpartum decreased the risk of LRTIs in the child up to age 5 y [8]. In our current study, we analyzed only infections with a confirmed viral pathogen within the first 36 mo, which limited the statistical power. Although there was a tendency toward a lower frequency of some viral infections, overall, no statistically significant association was observed for specific pathogens. Additional findings from the COPSAC**_2010_** trial have previously reported a reduced risk of gastroenteritis, a 2.5-day shorter duration of illness [10], and decreased risk of croup during the first 36 mo of life in the *n–3* LCPUFA group [9].

EPA and DHA may influence early life infection risk through several biological mechanisms. During pregnancy, fatty acids cross the placenta and are incorporated into fetal cell membranes, where they affect immune-cell signaling and precursor of proresolving mediators that help regulate inflammation [2]. These processes may make the effect of maternal intake of *n–3* LCPUFA more pronounced in early infancy (≤6 mo), when the infant’s own immune system is still immature, as supported by our findings. As children grow older, individual behaviors, environmental exposures, and dietary patterns likely become more important in determining infection risk, as supported by observational studies and randomized trials [[5], [6], [7],^27^,^28^]. Hakola et al. [28] found that higher serum levels of *n–3* LCPUFA in 576 children aged 6 to 18 mo were associated with reduced risk of CVB2 and RSV positivity at 18 mo, indicating that *n–3* LCPUFA may affect the risk of viral infections in early life. In the current study, we observed a weak inverse association between maternal *n*–3 LCPUFA intake and risk of CVB5 by age 6 mo in DIPP children, although not at 36 mo or for other CVBs. The number of children with CVB5 was low, which makes the result uncertain. Previous observations on CVB2 [28] and current CVB5 findings together support the possibility that *n–3* LCPUFAs may affect the risk of CVB infections in early childhood and that the risk may differ by type of virus. Finally, maternal fish oil supplementation has also been associated with immunologic changes in cord blood, which may persist and influence allergic sensitization and risk of asthma and allergic diseases [8,29,30]. However, the underlying mechanisms shaping immunological responses are complex and incompletely understood.

### Strengths and limitations

A major strength is that we could study the association of maternal *n–3* LCPUFAs intake and childhood infections in 3 Nordic cohorts, including one intervention trial, which complement each other. MoBa is a large, population-based prospective cohort with extensive questionnaire data collected prospectively during pregnancy and childhood. As in all longitudinal cohorts with intensive follow-up, participation was below 100% in both the cohorts and trial, but retention during the first 3 y was generally high [8,[12], [13], [14]]. Although <100% participation rate may raise concerns about selection bias, previous analyses demonstrated that exposure-outcome associations were not necessarily influenced by selective participation by comparing the MoBa cohort and Norwegian mothers giving birth during the same period [31]. In both MoBa and DIPP, maternal *n–3* LCPUFA intake from both food and supplements was assessed using validated FFQs. Nevertheless, dietary intakes were self-reported and may not fully capture individual variability. Therefore, biomarker data from the MoBa subcohort allowed for the objective evaluation of circulating DHA and EPA levels. The randomized trial design of the COPSAC**_2010_** study reduces confounding and strengthens causal inference. A strength of this trial is its unique longitudinal clinical follow-up, which included 9 visits during the first 36 mo of life and daily symptom diaries from birth until age 36 mo. However, the relatively small sample size of DIPP and COPSAC**_2010_** may limit their statistical power. The daily dose used in the COPSAC**_2010_** trial was much higher than the typical dietary intake in most populations, making it difficult to directly compare the results of the trial with observational studies.

The infection outcomes differed across the 3 studies. In MoBa, information on the infections was based on parental reports collected by questionnaires at 6, 18, and 36 mo of age. In DIPP, infection outcome was based on circulating neutralizing antibodies against 6 CVB serotypes, providing an objective and pathogen-specific measure. COPSAC**_2010_** used nasopharyngeal sampling following episodes of troublesome lung symptoms. Although this allowed direct viral detection, milder infections may have been missed.

The rich collection of data in the cohorts allowed us to adjust for a wide range of covariates, including vitamin D status. Adjustments for potential mediators, including breastfeeding, preterm birth, birth weight, and child’s own supplement use in MoBa, did not alter the associations, supporting a potential direct effect of maternal intake during pregnancy on the susceptibility to symptomatic infections in childhood.

In addition to study design, exposure levels, and endpoint definition, variation in other aspects of maternal diet (such as *n–6* PUFA levels) may have contributed to the inconsistent associations across cohorts. Furthermore, genetic factors that may influence both fatty-acid metabolism and susceptibility to childhood infections were not taken into account. Finally, EPA- and DHA-derived oxylipins, which are important mediators in immune and infection-related pathways, were not measured in our cohorts.

### Generalizability, interpretation, and clinical relevance

The study was done in Nordic countries in general population (MoBa) and among families with increased risk for type 1 diabetes (DIPP) and asthma (COPSAC), which may limit the generalization to other population groups. The findings of this study provide new information on the potential role of maternal *n–3* LCPUFA in reducing the occurrence of infections in early childhood. Intake levels of *n–3* LCPUFA varied across the cohorts, reflecting differences in dietary habits, including fish consumption and the use of supplements. Nordic children experience typically 3 to 4 respiratory infections and ≥ 1 episode of gastroenteritis annually during their first 3 y of life [32,33]. This suggests some clinical relevance, although the effect was small and mostly not statistically significant. Yet, the lack of consistent findings across all 3 cohorts underscores the complexity of the relationship between maternal diet and offspring health.

### Conclusions

In conclusion, this study does not provide consistent evidence that higher maternal *n–3* LCPUFA pregnancy intake reduces the risk of infections in early childhood. The intake levels of EPA and DHA and the dietary assessment methods used varied considerably across the included studies, making direct comparisons challenging. Nevertheless, inconsistencies across cohorts highlight the need for further studies to confirm these associations and clarify underlying mechanisms.

## Author contributions

The authors’ responsibilities were as follows – AKR, LH, and NB: conducted the analysis and drafted the manuscript; LCS, KB, and SMV: supervised the study; and all authors: contributed to the study design, interpretation of results, critically revised the manuscript, and approved the final version of the article.

## Data availability

Data from the Norwegian Mother, Father and Child Cohort Study is managed by the Norwegian Institute of Public Health. Access requires approval from the Regional Committees for Medical and Health Research Ethics (REC), compliance with GDPR, and data owner approval. Participant consent does not allow individual-level data storage in repositories or journals. Researchers seeking access for replication must apply via www.helsedata.no. The data used in the DIPP study is managed by Tampere University and the Finnish Institute for Health and Welfare. Individual-level data cannot be shared due to the sensitive nature of the data and the existing legislation. Data will be available with a signed data agreement approved for a specific purpose on request to nicklas.brustad@dbac.dk.

## Funding

This study has received funding from the European Union’s Horizon 2020 research and innovation program under grant agreement No 874864 HEDIMED. The Norwegian Mother, Father and Child Cohort Study is supported by the Norwegian Ministry of Health and Care Services and the Ministry of Education and Research, NIH/NIEHS (contract no N01-ES-75558), NIH/NINDS (grant no.1 UO1 NS 047537-01 and grant no.2 UO1 NS 047537-06A1). The Finnish Diabetes Prediction and Prevention Study was supported by JDRF/Breakthrough T1D (grants 1-SRA-2016-342-MR, 1-SRA-2019-732-M-B, 3-SRA-2020-955-S-B), European Commission (grant BMH4-CT98-3314), Novo Nordisk Foundation, Research Council of Finland (Decisions No 292538 and No 339922), Diabetes Research Foundation in Finland, and Special Research Funds for University Hospitals in Finland. N.B. received funding from The Lundbeck Foundation (R381-2021-1428) and from the Independent Research Fund Denmark (5253-00016B).

## Conflict of interest

The authors report no conflicts of interest.
