# Supplementary material for: Marine n–3 Long-Chain Polyunsaturated Fatty Acid Intake in Pregnancy and Risk of Early Life Infections in 3 Nordic Cohorts: A HEDIMED Consortium Study
Source: J Nutr. 2026 Feb 28;156(5):101456. doi: 10.1016/j.tjnut.2026.101456 (PMC13197902; doi:10.1016/j.tjnut.2026.101456)
Supplement: Multimedia component 1 [file mmc1.docx]

**Supplementary material to:**

**Marine *n-3* long-chain polyunsaturated fatty acid intake in pregnancy and risk of early life infections: a HEDIMED consortium study**

Aino K. Rantala^1,2^*, Leena Hakola^3,4,5^*, Nicklas Brustad^6^*, German Tapia^1^, Elin M. Hård Af Segerstad^7,8^, Jussi Lehtonen^9^, Jonathan Thorsen^6,10^, Mari Åkerlund^3,4^, Christine L. Parr^11^, Maria C. Magnus^12^, Nicolai A. Lund-Blix^1,13^, Jakob Stokholm^6,14^, Mikael Knip^15,16^, Jorma Toppari^17,18^, Ketil Størdal^7,19^, Riitta Veijola^20,21^, Heikki Hyöty^9,22,23^, Suvi M. Virtanen^3,4,5,24^**, Klaus Bønnelykke^6,10^**, Lars C. Stene^1^**, and the HEDIMED Investigator Group

Contents

[**Supplementary methods – details of dietary methods in MoBa and DIPP** 3](#_Toc221790863)

[**Supplementary result for the MoBa study** 4](#_Toc221790864)

[Supplementary Figure 1. Flow chart showing formation of the MoBa study analysis sample 4](#_Toc221790865)

[Supplementary Table 1. Characteristics of the MoBa study participants at 6 and 36-month follow-ups 5](#_Toc221790866)

[Supplementary Table 2. Association between total intake of *n*-3 LCPUFA (g/day) in pregnancy and number of LRTIs, URTIs and gastroenteritis during childhood in the MoBa study. 7](#_Toc221790867)

[Supplementary Table 3. Association between total intake of *n*-3 LCPUFA (g/day) in pregnancy and doctor visit or hospitalization for LRTI, URTI and gastroenteritis during childhood in the MoBa study 8](#_Toc221790868)

[Supplementary Table 4. Association between total intake of *n*-3 LCPUFA (g/day) in pregnancy and number of croup episodes during childhood in the MoBa study 9](#_Toc221790869)

[Supplementary Table 5. Association between measured DHA and EPA fatty acids in phospholipid fraction of whole blood collected at mid-pregnancy and number of LRTIs, URTIs and gastroenteritis during childhood in the MoBa subcohort (n=976) 9](#_Toc221790870)

[**Supplementary results for the DIPP study** 10](#_Toc221790871)

[Supplementary Table 6. DIPP participant characteristics for children. 10](#_Toc221790872)

[Supplementary Table 8. Total intake of *n*-3 LCPUFA during pregnancy and odds for children of having an additional CVB infection by age 36 months in the DIPP study* 12](#_Toc221790873)

# **Supplementary methods – details of dietary methods in MoBa and DIPP**

Detailed description of the food frequency questionnaire (FFQ) methods used to quantify pregnancy intake of *n*-3 LCPUFAs in DIPP and MoBa

*MoBa sub-cohort with measured maternal EPA and DPA in blood samples from pregnancy*

The relative amount of Eicosapentaenoic acid (EPA) and docosahexaenoic acid (DHA) in whole blood phospholipid fractions measured in gestational week 18 in a sub cohort of 957 pregnant women in the Norwegian Mother, Father, and Child cohort study giving birth during 2002-2003(4). Venous non-fasting EDTA whole blood sample were collected in mid-pregnancy at median gestational week 18 and sent to the MoBa biobank in Oslo. Whole blood sample were stored at -80℃ until analysis (5). EPA and DHA were measured in the phospholipid fraction of whole blood at Vitas Analytical Services in Oslo (https://www.vitas.no/), Norway (VITAS method AM-042, [www.vitas.no](file:///C:\Users\ELSEGE\OneDrive%20-%20Lund%20University\Forskning\Postdoc%20Olso\Projekt\Fettsyror%20och%20celiaki\Manuscript\Första%20runda%20medförfattare\www.vitas.no)). EPA and DHA (and other fatty acids not considered here) were measured in the phospholipid fraction after solid-phase extraction, and gas chromatography with flame-ionizing detection (GS-FID, Agilent 7890A, Agilent Technologies, Palo Alto, CA, USA). Relative amounts were used in the statistical analyses (% weight of total phospholipid fatty acids). We compared the FFQ estimated intakes of the sum total of EPA and DHA against the sum of DHA and EPA in the blood.

The correlation between the total sum intake of EPA and DHA from food and supplement from the MoBa FFQ was correlated with the relative amount of EPA and DHA in whole blood phospholipid fraction, with a Pearson coefficient of correlation of 0.51 (Hård af Segerstad et al., Manuscript in preparation/submitted).

# **Supplementary result for the MoBa study**


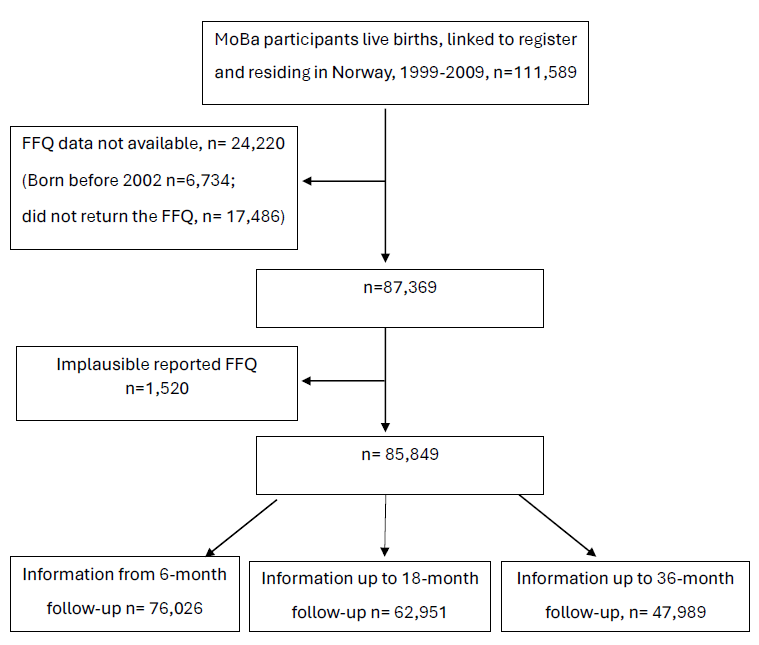


Supplementary Figure 1. Flow chart showing formation of the MoBa study analysis sample Participants with an implausible reported energy intake (<4.5 megajoules or >20 megajoules), or an incomplete FFQ (defined as four or more blank pages) were excluded. FFQ, food frequency questionnaire; MoBa, Norwegian Mother, Father and Child Cohort Study; n, number

## Supplementary Table 1. Characteristics of the MoBa study participants at 6 and 36-month follow-ups

| **Characteristics** | **MoBa at 6 months**  **N (%)** | **MoBa at 36 months**  **N (%)** |
| --- | --- | --- |
| **Total** | **76,026 (100.0)** | **47,989 (100)** |
| ***Maternal age, years*** |  |  |
| < 25 | 7,389 (9.7) | 3,952 (8.2) |
| 25-29 | 25,041 (32.9) | 15,795 (32.9) |
| 30-34 | 30,012 (39.5) | 19,446 (40.5) |
| ≥ 35 | 13,584 (17.9) | 8,796 (18.3) |
| ***Maternal parity*** |  |  |
| 0-1 | 61,892 (81.4) | 39,547 (82.4) |
| 2 or more | 14,134 (18.6) | 8,442 (17.6) |
| ***Maternal pre-pregnancy BMI*** |  |  |
| < 30 | 67,006 (88.1) | 42,764 (89.1) |
| Obese ≥ 30 | 6,924 (9.1) | 4,126 (8.6) |
| missing | 2,096 (2.8) | 1,099 (2.3) |
| ***Maternal education*** |  |  |
| Less than high school/high school | 49,489 (65.1) | 32,939 (68.6) |
| College | 25,892 (34.1) | 14,765 (30.8) |
| Missing | 645 (0.9) | 285 (0.6) |
| ***Maternal asthma (before or during pregnancy)*** |  |  |
| No | 70,110 (92.2) | 44,462 (92.7) |
| Yes | 5,578 (7.3) | 3,414 (7.1) |
| missing | 338 (0.5) | 113 (0.2) |
| ***Maternal smoking during pregnancy**** |  |  |
| Never | 54,975 (72.3) | 35,865 (74.7) |
| Previous | 4,180 (5.5) | 2,578 (5.4) |
| Stopped by 18 gestational weeks | 10,206 (13.4) | 5,981 (12.5) |
| Continued after 18 gestational weeks | 6,109 (8.0) | 3,296 (6.9) |
| Missing | 556 (0.7) | 269 (0.6) |
| ***Child sex*** |  |  |
| Male | 38,850 (51.1) | 24,457 (51.0) |
| Female | 37,176 (48.9) | 23,532 (49.0) |
| ***Birth year*** |  |  |
| 2002 | 3,911 (5.1) | 2,559 (5.3) |
| 2003 | 10,442 (13.7) | 6,866 (14.3) |
| 2004 | 11,065 (14.6) | 7,059 (14.7) |
| 2005 | 12,140 (16.0) | 7,719 (16.1) |
| 2006 | 13,568 (17.9) | 8,457 (17.6) |
| 2007 | 12,233 (16.1) | 7,485 (15.6) |
| 2008 | 10,162 (13.4) | 6,255 (13.0) |
| 2009 | 2,505 (3.3) | 1,589 (3.3) |
| ***Total energy intake (KJ) during pregnancy*** |  |  |
| Median (IQR) | 9,357 (7,908-11,119) | 9,329 (7,911-11,028) |
| Mean (SD) | 9,696.8 (2,541.2) | 9,651.6 (2,478.6) |
| ***Intake of n-3 LCPUFA (g/day) during pregnancy*** |  |  |
| Median (IQR) | 0.6 (0.3-1.1) | 0.6 (0.3-1.2) |
| Mean (SD) | 0.8 (0.7) | 0.8 (0.7) |
| ***Intake of n-3 LCPUFA from the diet (g/day)*** |  |  |
| Median (IQR) | 0.3 (0.2-0.5) | 0.3 (0.2-0.5) |
| Mean (SD) | 0.4 (0.4) | 0.4 (0.4) |
| ***Intake of n-3 LCPUFA from the supplements (g/day)*** ***among users*** | No. of users = 52,211 (68.7%) | No. of users = 33,798 (70.4%) |
| Median (IQR) | 0.3 (0.2-0.8) | 0.3 (0.2-0.8) |
| Mean (SD) | 0.6 (0.6) | 0.6 (0.6) |

BMI, body mass index; g, gram; IQR, interquartile range; KJ, kilojoule; MoBa, Norwegian Mother, Father and Child Cohort Study; N, number; SD, standard deviation;

## Supplementary Table 2. Association between total intake of *n*-3 LCPUFA (g/day) in pregnancy and number of LRTIs, URTIs and gastroenteritis during childhood in the MoBa study.

|  | **0-6 months, n= 76,026** | | **0-18 months, n=72,665** | | **0-36 months, n= 47,989** | |
| --- | --- | --- | --- | --- | --- | --- |
|  | **crude IRR (95% CI)** | **adj. IRR (95% CI)^a^** | **crude IRR (95% CI)** | **adj. IRR (95% CI)^a^** | **crude IRR (95% CI)** | **adj. IRR (95% CI) ^a^** |
| ***Lower respiratory tract infection (LRTI)*** | | | | | | |
| *Food and supplement* | 0.94 (0.90-0.99) | 0.96 (0.90-1.02) | 0.97 (0.94-1.00) | 0.98 (0.94-1.02) | 0.98 (0.95-1.01) | 0.99 (0.94-1.03) |
| Supplement | 0.89 (0.84-0.95) | 0.92 (0.85-0.99) | 0.95 (0.91-0.99) | 0.98 (0.93-1.04) | 0.95 (0.91-0.99) | 0.97 (0.92-1.03) |
| Food | 1.04 (0.95-1.13) | 1.01 (0.92-1.11) | 1.00 (0.95-1.06) | 0.98 (0.92-1.04) | 1.03 (0.97-1.09) | 1.00 (0.94-1.06) |
| *Adj. for mediators^b^* |  |  |  |  |  |  |
| *Food and supplement* |  | 0.97 (0.91-1.04) |  | 0.98 (0.95-1.03) |  | 1.00 (0.97-1.04) |
| Supplement |  | 0.94 (0.87-1.01) |  | 0.99 (0.94-1.05) |  | 0.97 (0.91-1.03) |
| Food |  | 1.02 (0.92-1.12) |  | 0.98 (0.92-1.05) |  | 1.00 (0.94-1.07) |
| ***Upper respiratory tract infection (URTI)*** | | | | | | |
| *Food and supplement* | 0.98 (0.97-0.99) | 0.97 (0.96-0.98) | 0.99 (0.98-1.00) | 0.99 (0.98-0.99) | 0.99 (0.99-1.00) | 0.99 (0.98-0.99) |
| Supplement | 0.97 (0.96-0.98) | 0.96 (0.95-0.98) | 1.00 (0.99-1.01) | 0.99 (0.98-1.00) | 0.99 (0.99-1.00) | 0.98 (0.97-0.99) |
| Food | 1.00 (0.98-1.01) | 0.98 (0.96-1.00) | 0.99 (0.97-1.00) | 0.98 (0.97-1.00) | 1.00 (0.99-1.01) | 0.99 (0.98-1.00) |
| *Adj. for mediators^b^* |  |  |  |  |  |  |
| *Food and supplement* |  | 0.97 (0.96-0.99) |  | 0.98 (0.98-0.99) |  | 0.99 (0.98-1.00) |
| Supplement |  | 0.97 (0.95-0.98) |  | 0.99 (0.98-1.00) |  | 0.98 (0.97-0.99) |
| Food |  | 0.98 (0.96-1.00) |  | 0.99 (0.98-1.00) |  | 0.99 (0.98-1.00) |
| ***Gastroenteritis*** |  |  |  |  |  |  |
| *Food and supplement* | 0.96 (0.93-0.99) | 0.94 (0.91-0.98) | 0.99 (0.98-1.00) | 0.97 (0.96-0.99) | 1.00 (0.99-1.01) | 0.96 (0.95-0.98) |
| Supplement | 0.94 (0.91-0.98) | 0.95 (0.90-1.00) | 1.00 (0.98-1.01) | 0.98 (0.97-1.00) | 1.01 (0.99 (1.02) | 0.96 (0.95-0.98) |
| Food | 0.98 (0.93-1.04) | 0.94 (0.89-1.00) | 0.98 (0.96-1.00) | 0.96 (0.94-0.98) | 0.98 (0.97-1.00) | 0.96 (0.94-0.98) |
| *Adj for mediators^b^* |  |  |  |  |  |  |
| *Food and supplement* |  | 0.96 (0.93-1.00) |  | 0.98 (0.96-0.99) |  | 0.96 (0.95-0.98) |
| Supplement |  | 0.96 (0.91-1.01) |  | 0.99 (0.97-1.01) |  | 0.97 (0.95-0.98) |
| Food |  | 0.96 (0.90-1.03) |  | 0.97 (0.94-0.99) |  | 0.96 (0.94-0.98) |

Adj, Adjusted; CI, confidence interval; IRR, incidence rate ratio; LCPUFA, long-chain polyunsaturated fatty acids; MoBa, Norwegian Mother, Father and Child Cohort Study; n, number; ^a^ Adjusted for maternal characteristics such as age at delivery, parity, educational level, smoking during pregnancy, asthma, total energy intake in pregnancy, vitamin D intake from food and supplement during pregnancy, and pre-pregnancy body mass index, as well as child characteristic such as sex, year and month of birth, country of birth, and county of residence. ^b^ In addition, adjusted for potential mediators: still breastfed at 6 months, preterm birth, birth weight, and child own fatty acid supplementation at 6/18 months.

## Supplementary Table 3. Association between total intake of *n*-3 LCPUFA (g/day) in pregnancy and doctor visit or hospitalization for LRTI, URTI and gastroenteritis during childhood in the MoBa study

|  | 0-6 months, n=76,026 | | 0-18 months, n=72,665 | | 0-36 months, n= 47,989 | |
| --- | --- | --- | --- | --- | --- | --- |
|  | **crude RR (95% CI)** | **adj. RR (95% CI)^a^** | **crude RR (95% CI)** | **adj. RR (95% CI) ^a^** | **crude RR (95% CI)** | **adj. RR (95% CI) ^a^** |
| ***Lower respiratory tract infection (LRTI)*** | | | | | | |
| *Food and supplement* | 0.94 (0.89-0.98) | 0.95 (0.89-1.01) | 0.96 (0.92-1.00) | 0.98 (0.93-1.03) | 0.96 (0.92-1.00) | 0.96 (0.91-1.03) |
| Supplement | 0.89 (0.84-0.95) | 0.92 (0.85-0.98) | 0.94 (0.89-0.99) | 0.97 (0.91-1.04) | 0.95 (0.90-1.00) | 0.95 (0.88-1.03) |
| Food | 1.02 (0.94-1.10) | 1.00 (0.92-1.08) | 1.00 (0.93-1.07) | 0.99 (0.92-1.07) | 0.97 (0.90-1.05) | 0.97 (0.99-1.06) |
| *Adj. for mediators^b^* |  |  |  |  |  |  |
| *Food and supplement* |  | 0.96 (0.87-1.05) |  | 1.00 (0.95-1.05) |  | 0.97 (0.91-1.04) |
| Supplement |  | 0.94 (0.87-1.01) |  | 0.99 (0.93- 1.06) |  | 0.96 (0.88-1.04) |
| Food |  | 1.01 (0.93-1.09) |  | 1.01 (0.93-1.09) |  | 0.99 (0.91-1.07) |
| ***Upper respiratory tract infection (URTI)*** | | | | | | |
| *Food and supplement* | 0.96 (0.95-0.98) | 0.98 (0.96-1.01) | 0.97 (0.95-0.99) | 1.00 (0.97-1.03) | 0.98 (0.96-0.99) | 1.01 (0.98-1.04) |
| Supplement | 0.94 (0.92-0.96) | 0.98 (0.95-1.01) | 0.95 (0.93-0.97) | 0.99 (0.95-1.02) | 0.95 (0.93-0.97) | 0.99 (0.96-1.03) |
| Food | 1.00 (0.97-1.03) | 0.99 (0.96-1.02) | 1.01 (0.98-1.05) | 1.01 (0.98-1.05) | 1.02 (0.99-1.06) | 1.02 (0.99-1.06) |
| *Adj. for mediators^b^* | *Models don’t converge* | |  |  |  |  |
| ***Gastroenteritis*** |  |  |  |  |  |  |
| *Food and supplement* | 1.00 (0.95-1.06) | 1.00 (0.94-1.06) | 0.99 (0.94-1.04) | 0.99 (0.93-1.04) | 1.00 (0.95-1.05) | 0.92 (0.86-0.99) |
| Supplement | 1.04 (0.97-1.12) | 1.04 (0.96-1.12) | 1.00 (0.93-1.07) | 1.00 (0.93-1.08) | 0.99 (0.93-1.06) | 0.91 (0.83-1.01) |
| Food | 0.91 (0.82-1.02) | 0.92 (0.82-1.04) | 0.95 (0.86-1.05) | 0.95 (0.85-1.06) | 0.96 (0.87-1.06) | 0.91 (0.82-1.01) |
| *Adj. for mediators^b^* |  |  |  |  |  |  |
| *Food and supplement* |  | 1.02 (0.96-1.09) |  | 1.01(0.95 -1.06) |  | 0.93 (0.87-1.00) |
| Supplement |  | 1.06 (0.98-1.15) |  | 1.02 (0.95-1.09) |  | 0.93 (0.84-1.02) |
| Food |  | 0.94 (0.83-1.05) |  | 0.96 (0.87-1.07) |  | 0.92 (0.83-1.02) |

Adj, Adjusted; CI, confidence interval; LCPUFA, long-chain polyunsaturated fatty acids; MoBa, Norwegian Mother, Father and Child Cohort Study; n, number; RR, risk ratio;

^a^ Adjusted for maternal characteristics such as age at delivery, parity, educational level, smoking during pregnancy, asthma, total energy intake in pregnancy, vitamin D intake from food and supplement during pregnancy, and pre-pregnancy body mass index, as well as child characteristic such as sex, year and month of birth, country of birth, and county of residence.

^b^ In addition, adjusted for potential mediators: still breastfed at 6 months, preterm birth, birth weight, and child own fatty acid supplementation at 6/18 months.

## Supplementary Table 4. Association between total intake of *n*-3 LCPUFA (g/day) in pregnancy and number of croup episodes during childhood in the MoBa study

|  | **0-6 months, n**= **76,026** | | **0-18 months, n=72,665** | | **0-36 months, n=52,453** | |
| --- | --- | --- | --- | --- | --- | --- |
|  | **crude IRR (95% CI)** | **adj. IRR (95% CI) ^a^** | **crude IRR (95% CI) ^a^** | **adj. IRR (95% CI) ^a^** | **crude IRR (95% CI) ^a^** | **adj. IRR (95% CI) ^a^** |
| **Food and supplement** | 0.94 (0.87-1.02) | 0.95 (0.85-1.06) | 1.01 (0.97-1.06) | 1.01 (0.96-1.07) | 1.01 (0.97-1.05) | 1.02 (0.97-1.08) |
| Supplement | 0.92 (0.83-1.02) | 0.96 (0.82-1.11) | 0.94 (0.89-0.99) | 0.94 (0.87-1.01) | 0.96 (0.92-1.01) | 0.98 (0.91-1.05) |
| Food | 0.99 (0.86-1.14) | 0.94 (0.80-1.09) | 1.16 (1.08-1.25) | 1.11 (1.03-1.20) | 1.11 (1.03-1.19) | 1.08 (1.00-1.16) |

Adj, Adjusted; CI, confidence interval; IRR, incidence rate ratio; LCPUFA, long-chain polyunsaturated fatty acids; MoBa, Norwegian Mother, Father and Child Cohort Study; n, number

^a^ Adjusted for maternal characteristics such as age at delivery, parity, educational level, smoking during pregnancy, asthma, total energy intake in pregnancy, vitamin D intake from food and supplement during pregnancy, and pre-pregnancy body mass index, as well as child characteristic such as sex, year and month of birth, country of birth, and county of residence.

^b^ In addition adjusted for potential mediators: breastfeeding still at 6 months, preterm birth, birth weight, and child own fatty acid supplementation

Supplementary Table 5. Association between measured DHA and EPA fatty acids in phospholipid fraction of whole blood collected at mid-pregnancy and number of LRTIs, URTIs and gastroenteritis during childhood in the MoBa subcohort (n=976)

|  | **0-6 months** | | **0-18 months** | | **0-36 months** | |
| --- | --- | --- | --- | --- | --- | --- |
|  | **crude IRR (95% CI)** | **adj. IRR (95% CI)^a^** | **crude IRR (95% CI)** | **adj. IRR (95% CI) ^a^** | **crude IRR (95% CI)** | **adj. IRR (95% CI) ^a^** |
| **LRTIs** | 0.93 (0.79-1.08) | 0.93 (0.77-1.10) | 0.99 (0.90-1.08) | 0.99 (0.88-1.10) | 0.95 (0.87-1.03) | 0.97 (0.88-1.08) |
| **URTIs** | 1.00 (0.97-1.04) | 0.99 (0.95-1.03) | 1.00 (0.97-1.02) | 1.01 (0.98-1.04) | 0.99 (0.98-1.01) | 1.01 (0.99-1.03) |
| **Gastroenteritis** | 0.94 (0.83-1.07) | 0.97 (0.84-1.13) | 0.98 (0.93-1.02) | 0.99 (0.93-1.04) | 0.98 (0.95-1.01) | 0.99 (0.96-1.03) |

Adj, Adjusted; CI, confidence interval; DHA, docosahexaenoic acid; EPA, eicosapentaenoic acid; IRR, incidence rate ratio; MoBa, Norwegian Mother, Father and Child Cohort Study; n, number

^a^ Adjusted for maternal characteristics such as age at delivery, parity, educational level, smoking during pregnancy, asthma, total energy intake in pregnancy, vitamin D intake from food and supplement during pregnancy, and pre-pregnancy body mass index, as well as child characteristic such as sex, year and month of birth, country of birth, and county of residence.

# **Supplementary results for the DIPP study**

## Supplementary Table 6. DIPP participant characteristics for children.

|  | **N 560** | **% or**  **mean (Std. Dev.)** |
| --- | --- | --- |
| **Sex** |  |  |
| Female | 214 | 43.6 |
| Male | 346 | 56.4 |
| **Case-control status** |  |  |
| Case (with type 1 diabetes-related autoimmunity) | 184 | 67.1 |
| Control | 376 | 32.9 |
| **Hospital of birth** |  |  |
| Tampere | 316 | 56.4 |
| Oulu | 244 | 43.6 |
| **Number of siblings at birth** |  |  |
| 0 | 264 | 47.1 |
| 1 | 171 | 30.5 |
| 2 | 74 | 13.2 |
| ≥3 | 36 | 6.4 |
| Missing | 15 | 2.7 |
| **Smoking during pregnancy** |  |  |
| No | 511 | 91.3 |
| Yes | 36 | 6.4 |
| Missing | 18 | 2.3 |
| **Birth year** |  |  |
| 1997 | 8 | 1.4 |
| 1998 | 100 | 17.9 |
| 1999 | 110 | 19.6 |
| 2000 | 83 | 14.8 |
| 2001 | 71 | 12.7 |
| 2002 | 55 | 9.8 |
| 2003 | 86 | 15.4 |
| 2004 | 47 | 8.4 |
| **Maternal age at the time of the child´s birth** | 560 | 30.0 (5.4) |
| **Estimated pre-pregnancy BMI** | 531 | 24.5 (4.4) |

DIPP, The Finnish Type 1 Diabetes Prediction and Prevention

Supplementary Table 7. Total intake of *n*-3 LCPUFA during pregnancy and risk of CVB infection in the children by age 6, 18 and 36 months in the DIPP study*

|  | **CVB No** | **CVB Yes** | **Unadjusted** |  | **CVB No** | **CVB**  **Yes** | **Adjusted^a^** |  |
| --- | --- | --- | --- | --- | --- | --- | --- | --- |
| **Virus** | **n** | **n** | **Odds Ratio (95%CI)** | **P** | **n** | **n** | **Odds Ratio (95%CI)** | **P** |
| **CVB infections at age 0-6 months** | | | | | | | | |
| CVB1 | 375 | 147 | 0.73 (0.29, 1.86) | 0.51 | 342 | 134 | 0.57 (0.20, 1.68) | 0.31 |
| CVB2 | 443 | 46 | 0.97 (0.24., 3.89) | 0.97 | 405 | 41 | 1.43 (0.34, 6.05) | 0.63 |
| CVB3 | 485 | 38 | 1.53 (0.41, 5,69) | 0.53 | 442 | 35 | 1.23 (0.26, 5.87) | 0.79 |
| CVB4 | 480 | 44 | 1.49 (0.42, 5.20) | 0.53 | 437 | 40 | 1.22 (0.29, 5.09) | 0.79 |
| CVB5‡ | 485 | 11 | 0.006 (0.00, 1.31) | 0.063 | 444 | 9 | 0.00 (0.00, 0.13) | 0.018 |
| CVB6 | 478 | 9 | 0.49 (0.13, 19.2) | 0.71 | 436 | 8 | 1.28 (0.07, 24.9) | 0.87 |
| Any CVB | 287 | 199 | 0.76 (0.33, 1.77) | 0.52 | 263 | 180 | 0.63 (0.24, 1.64) | 0.34 |
| **CVB infections at 0-18 months** | | | | | | | | |
| CVB1 | 319 | 237 | 0.64 (0.28, 1.14) | 0.28 | 286 | 219 | 0.72 (0.30, 1.73) | 0.46 |
| CVB2 | 369 | 127 | 1.12 (0.46, 2.71) | 0.80 | 363 | 114 | 1.65 (0.62, 4.36) | 0.32 |
| CVB3 | 491 | 65 | 2.88 (1.09, 7.65) | 0.033 | 444 | 61 | 2.55 (0.86, 7.56) | 0.092 |
| CVB4 | 488 | 68 | 1.28 (0.43, 3.80) | 0.66 | 444 | 61 | 1.37 (0.43, 4.41) | 0.560 |
| CVB5 | 509 | 20 | 0.06 (0.002, 1.70) | 0.10 | 466 | 17 | 0.01 (0.000, 1.28) | 0.064 |
| CVB6 | 490 | 33 | 0.38 (0.05, 2.92) | 0.35 | 447 | 30 | 0.39 (0.04, 3.61) | 0.41 |
| Any CVB | 192 | 331 | 1.11 (0.49, 2.50) | 0.81 | 177 | 300 | 1.50 (0.61, 3.69) | 0.37 |
| **CVB infections at 0-36 months** | | | | | | | | |
| CVB1 | 301 | 257 | 0.55 (0.24, 1.24) | 0.15 | 269 | 238 | 0.58 (0.24, 1.43) | 0.24 |
| CVB2 | 376 | 173 | 1.48 (0.67, 3.29) | 0.34 | 340 | 158 | 1.85 (0.77, 4.45) | 0.17 |
| CVB3 | 483 | 75 | 2.31 (0.90, 5.95) | 0.083 | 436 | 71 | 1.98 (0.69, 5.67) | 0.20 |
| CVB4 | 480 | 78 | 1.28 (0.46, 3.59) | 0.64 | 439 | 68 | 1.38 (0.46, 4.16) | 0.56 |
| CVB5 | 524 | 28 | 0.22 (0.02, 2.50) | 0.22 | 477 | 24 | 0.09 (0.004, 1.92) | 0.12 |
| CVB6 | 484 | 65 | 1.11 (0.35, 3.50) | 0.86 | 438 | 60 | 1.32 (0.41, 4.27) | 0.64 |
| Any CVB | 157 | 392 | 1.25 (0.52, 3.03) | 0.62 | 144 | 354 | 1.74 (0.64, 4.72) | 0.28 |

DIPP, The Finnish Type 1 Diabetes Prediction and Prevention LCPUFA, long-chain polyunsaturated fatty acids

* *n*-3 LCPUFA intake g/d is calculated from food frequency questionnaires, and modelled as continuous variable in logistic regression with binary outcome coxsackievirus B (CVB) 1-6, and any CVB yes/no.

**^a^** Adjusted for islet autoimmunity case-control status, sex, birth year, birth month, birth area, number of siblings, smoking, and pre-pregnancy body mass index (BMI).

‡ Estimates for association with CVB 5 after rescaling the DHA+EPA intake to mg/d: Unadjusted OR 0.995 (0.990, 1.000); Adjusted OR 0.988 (0.979, 0.998) (p-values are unaffected by rescaling). These results should be interpreted with caution due to relatively small number with CVB5 infection.

## Supplementary Table 8. Total intake of *n*-3 LCPUFA during pregnancy and odds for children of having an additional CVB infection by age 36 months in the DIPP study*

| **CVB infections** | **Unadj. OR (95% CI)** | **P** | **Adjusted OR^a^ (95% CI)** | **P** |
| --- | --- | --- | --- | --- |
| by 6 mo | 0.79 (0.35, 1.79) | 0.57 | 0.68 (0.27, 1.69) | 0.40 |
| by 18 mo | 1.05 (0.51, 2.13) | 0.90 | 1.33 (0,62, 2.86) | 0.46 |
| by 36 mo | 1.27 (0.64, 2.31) | 0.50 | 1.49 (0.70, 3.14) | 0.30 |

DIPP, The Finnish Type 1 Diabetes Prediction and Prevention LCPUFA, long-chain polyunsaturated fatty acids

* Sum intake of docosahexaenoic acid (DHA; C22:6, n-3) and eicosatetraenoic acid (EPA, C20:5, n-3) in g/d estimated from food frequency questionnaires, and modelled as continuous independent variable in ordinal logistic regression with outcome being the number of different coxsackievirus B (CVB) serotypes among CVB1-6 being positive in serum by age 36 months in the children. OR: Odds ratio. CI, confidence interval;

^a^ Adjusted for case-control status, sex, birth year, birth month, birth area, number of siblings, smoking, pre pregnancy body mass index (BMI).
